# Bowel obstruction from wild bananas: a neglected health problem in Laos

**DOI:** 10.1258/td.2011.100293

**Published:** 2011-04

**Authors:** Günther Slesak, Kaisouksavanh Mounlaphome, Saythong Inthalad, Ounheaun Phoutsavath, Mayfong Mayxay, Paul N Newton

**Affiliations:** * SFE Medical Project, Luang Namtha, Lao PDR; † Tropenklinik Paul-Lechler-Krankenhaus, Tübingen, Germany; ‡ Luang Namtha Provincial Hospital, Luang Namtha, Lao PDR; § Wellcome Trust-Mahosot Hospital-Oxford Tropical Medicine Research Collaboration, Microbiology Laboratory, Mahosot Hospital, Vientiane, Lao PDR; ** Centre for Clinical Vaccinology and Tropical Medicine, Churchill Hospital, University of Oxford, Oxford, UK; †† Faculty of Postgraduate Studies, University of Health Sciences, Vientiane, Lao PDR

## Abstract

We investigated the significance and risk factors of bowel obstruction caused by the consumption of wild bananas (BOWB) in Laos. Of six patients with BOWB in Luang Namtha, North Laos, five required enterotomy for phytobezoars. All had eaten wild banana (WB) seeds.

Of 227 other patients/relatives: 91.2% had eaten WB; 46.3% had also eaten the seeds and 45.4% knew of complications resulting from eating WB; 42.3% were aware of the complications of ingesting the seeds (constipation [37.9%], appendicitis/abdominal pain/vomiting [2.6% each] and bloated stomach/death [1.3% each]). Middle/highland Lao ethnicity was associated with WB and seed consumption (odds ratio [OR] 9.91 and 2.33), male sex with WB consumption and unawareness (OR 4.31 and 1.78).

At all surgically-equipped hospitals in Laos, 33/44 doctors knew of BOWB, describing patients as young adults (16/30), male (24/30) and from middleland Lao (18/30). Countrywide, 46/48 patients with BOWB required laparotomy in 2009 (incidence 0.8/100,000). All consumed WB seeds. BOWB is widespread in Laos, especially among young middleland Lao men consuming WB seeds on an empty stomach.

## Background

Phytobezoars are unusual causes of bowel obstruction.^1^ Various fruits and vegetables have been reported to form these pathogenic concretions in the stomach or intestines. Persimmon (*Diospyros kaki*), orange (*Citrus spp.*) pith, grapefruit (*C. paradisi)*, mango (*Mangifera spp.*), carrots (*Daucus carota)*, pickled onions (*Allium cepa*) and green figs (*Ficus spp.*) have been reported.^1–8^ Bananas (*Musa spp.*) have rarely been recorded as causing phytobezoars^7^ but have been used to treat diarrhoea and dysentery^9,10^ and have been associated with vomiting and abdominal distension among neonates.^11^ Risk factors for phytobezoar formation include: previous gastric surgery; poor dentition; and increased fibre intake.^2,5,8^ In 2004 bezoars from wild bananas (WB) were described in four Lao patients, all of whom had no prior history of abdominal surgery.^12^ However, the geography, incidence and pathophysiology of this potentially life-threatening complication remain unclear. In 2009 three patients with bowel obstruction after wild banana consumption (BOWB) presented at Luang Namtha Provincial Hospital (LNPH) in a remote part of northern Laos. This province is a mountainous rural area, adjacent to China and Myanmar (Burma) with a population of 155,772 people. The majority belong to 17 of the country's 49 ethnic minorities, traditionally classified, according to their customary settlement, into three categories: lowland (Loum), middleland (Theung) and highland Lao (Soung).^13,14^ The Lao Loum represents 53% of the population.^14^ Laos is a low-income country (gross national income *per capita* US$580) and 33% live below the poverty line.^15^

**Figure 1:**
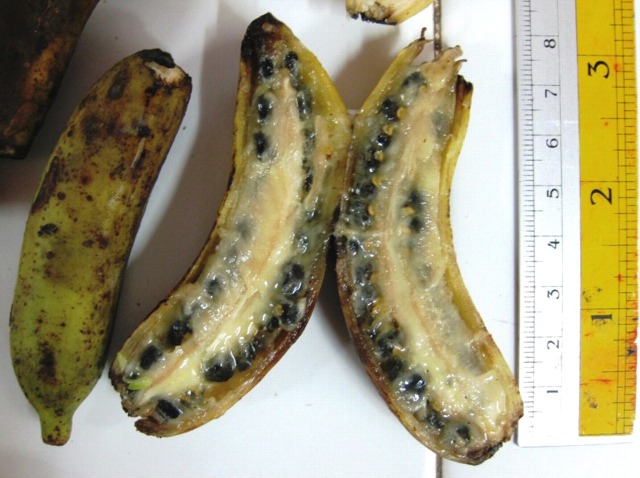
Wild banana *Musa acuminata* var. *chinensis* or var. *burmannica* from Luang Namtha, Laos

The natural range of WB extends throughout the Indo-Malaysian region, in the tropical and subtropical areas from Sri Lanka and eastern India, across south China and southeast Asia to the southwest Pacific and northern Australia.^10^ In northern Laos and the adjacent northern Thailand, Myanmar and China, *Musa acuminata* var. *chinensis* and var. *burmannica* have been recorded.^16^ Their ripe fruits taste sweet and astringent, contain numerous small seeds and are much smaller than the modern cultivars (Figure 1). We investigated possible risk factors of BOWB and its significance as a public health problem in Laos.

## Methods

### Patients

We describe six patients who had been diagnosed with BOWB between August 2008 and October 2009 at LNPH (4) and the Military Hospital (2), the only hospitals in Luang Namtha Province providing abdominal surgery. The patients were questioned about the circumstances of their banana consumption using a structured questionnaire and all gave their written informed consent.

### Hospital survey

During September–November 2009, after informed verbal consent was given, a sample of patients without BOWB, or their relatives, aged >17 years who were attending LNPH were interviewed using a structured questionnaire about their knowledge of the health problems associated with WB consumption. Data were anonymized.

### Country-wide telephone survey

During January–March 2010 we performed a telephone survey of surgeons, if they gave informed verbal consent, at all 38 hospitals in Laos that were potentially able to perform general abdominal surgery: provincial (*n* = 16), provincial military (*n* = 15), public referral (*n* = 2) and military referral (*n* = 5) hospitals. Abdominal surgery is not available in district hospitals. A structured interview included questions about incidence and associated factors of BOWB.

### Data analysis

Data were analysed with EpiInfo Version 3.4.3 (Centers for Disease Control and Prevention, GA, USA). Fisher's exact test and Student's *t*-test were used to compare categorical and continuous data, respectively. Gender, major ethnic group, education and profession were tested in univariate analysis. All factors with a *P* value ≤ 0.2 were then fitted into a multivariate logistic regression model. We considered *P* < 0.05 to be statistically significant. The minimum national incidence of BOWB requiring surgery was calculated as the total number of operated patients in 2009 with the total Lao population (∼6 million) as the denominator. In order to estimate the overall country-wide incidence of BOWB, we divided the incidence of operated patients by the rate of operations mentioned in the survey among patients and relatives at LNPH.

## Results

**Figure 2:**
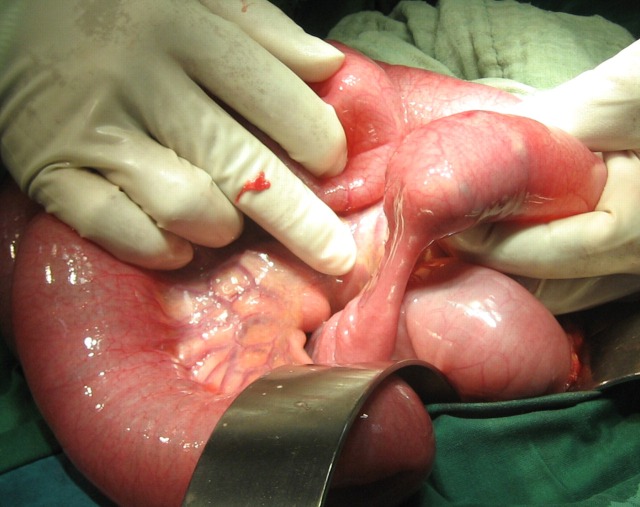
Intraoperative finding of patient no. 4 with a distended ileum proximal to one of the wild banana phytobezoars

**Figure 3:**
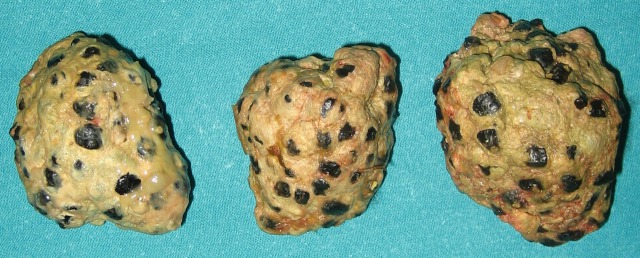
Wild banana phytobezoars from patient no. 4 (maximum diameter 5.0 cm)

The six patients with BOWB were aged 13–26 years: all had eaten WB when hungry while working in the fields; none had previous abdominal surgery; and all survived (Table 1). Obstruction occurred in the jejunum or ileum (Figures 2 and 3) for all but one (rectosigmoid). At LNPH the three operated patients represented 1.9% of all patients operated for acute/surgical abdomen (excluding gynaecological and obstetric cases) during the same period.

**Table 1:** Characteristics of six patients with bowel obstruction from wild bananas (BOWB)

Patients' data						Wild banana consumption (WBC)							Symptoms			Findings and treatment	
Patients' no.	Treating hospital	Date hospitalized	Age (years)	Sex	Ethnicity	No. of bananas	Ripeness and taste	Seeds eaten	Preceding fasting time (h)	Plus other food or drinks	Time until other food (h)	Time until drinks (h)	Interval between WBC and symptoms (days)	Abdominal pain/ distension + nausea and vomiting	Constipation (days)	No. of WB bezoars and location	Treatment
1	LNPH	August 2008	13	f	Khmu (middle Lao)	10	Ripe, sweet, astringent	Yes, all	5	No	2	2	30	+	3	1 before ileocoecal junction, size 6x3 cm	Ileotomy
2	MH	Summer 2009	26	f	Khmu (middle Lao)	No data	No data	Yes	No data	No data	No data	No data	No data	+	+	1 injejunum, 15 cm distal of stomach	Jejunotomy
3	MH	July 2009	16	m	Khmu (middle Lao)	30	Ripe, sweet, astringent	Yes, all	3	No	3	1	0.9	+	3	1 in smallintestine,size ofduck egg	Ileotomy
4	LNPH	August 2009	15	f	Akha (highland Lao)	6	Ripe, sweet, astringent	Yes, all	15	No	4	4	0.3	+	2	3; 2 instomach, 1 about 60 cmbeforeileocoecal junction,size ≤5 cm	Gastrotomy, ileotomy
5	LNPH	September 2009	21	m	Akha (highland Lao)	Often 25/day	Ripe, sweet, astringent	Yes, all	0-2	Variable	0-1	0-1	60	+	14	Impactedfaeces inrectum	Rectal enema
6	LNPH	October 2009	15	m	Khmu (middle Lao)	>10	Ripe, sweet, astringent	Yes, all	6	No	1	1	30	+	2	1; 20 cmbefore ileocoecal junction	Ileotomy
**Summary or median**	**4 LNPH, 2 MH**	**August 2008 until October 2009**	**15.5**	**m:f=3:3**	**4 middle, 2 highland Lao**	**>10**	**Ripe, sweet, astringent**	**Yes**	**5**	**No**	**2**	**1**	**30**	**+**	**3**	**5/6 had 1 in(distal)ileum**	**5/6 operated**

Note: Patients 4–6 were interviewed during the hospital stay; patient 1 was interviewed 1.5 years later and patient 3, together with the treating doctor, was interviewed 2 months later. Data for patient 2 were obtained only from the treating doctor about 7 months later as she had died in the meantime from an illness unrelated to the operation.

LNPH, Luang Namtha Provincial Hospital; MH, Military Hospital of Luang Namtha; WB, wild bananas

Of 227 patients and relatives interviewed at LNPH, 207 (91.2%) recalled eating WB, 105 (46.3%) recalled eating the seeds and 103 (45.4%) were aware of the possible associated health problems (Tables 2 and 3). Middleland and highland Lao ethnicity was associated with: previous consumption of WB (odds ratio [OR] 9.91, 95% confidence interval [CI] 2.2–44) and WB seeds (OR 2.33, 95% CI 1.4–4.0); being male with WB consumption; and an unawareness of the potential health consequences (OR 4.31 95% CI 1.5–12 and OR 1.78 95% CI 1.1–3.0, respectively, Tables 2 and 3). The consequences included: constipation/inability to pass stool in 86 (37.9%); appendicitis, abdominal pain, and vomiting (each in six, 2.6%); and swollen stomach or death after constipation (each in three, 1.3%); and dizziness in two (0.9%). Ninety-six (42.3%) blamed the banana seeds for these symptoms, including two who stated that eating seeds on an empty stomach was a contributory factor. Alarming modes of treatment were described, including the insertion of sticks and the piping of gasoline into the rectum. Only 12/103 (11.7%) stated that an operation was a potential remedy (Table 4).

**Table 2:** Factors associated with previous wild banana and banana seed consumption and an unawareness of health problems (univariate analysis) amongst patients (without bowel obstructions) and their relatives

		No. (%)	Odds ratio	95% confidence interval	P
Previous wild banana consumption (*n* = 204)	Male sex*	112 (54.9)	4.11	1.39–14.8	0.0036
	Middle/highland Lao ethnicities*	99 (48.8)	9.45	2.20–85.5	0.0002
	Illiteracy*	48 (23.6)	1.75	0.48–9.72	0.076
	Farmer	144 (70.6)	0.96	0.29–2.78	0.58
Previous wild banana seed consumption (*n* = 105)	Male sex	57 (54.3)	1.07	0.61–1.88	0.46
	Middle/highland Lao ethnicities*	59 (56.7)	2.33	1.31–4.18	0.0016
	Illiteracy*	28 (26.7)	1.41	0.71–2.78	0.18
	Farmer	77 (73.3)	1.18	0.63–2.24	0.34
Unawareness of wild banana health problems (*n* = 124)	Male sex*	72 (58.1)	1.78	1.02–3.13	0.021
	Lowland Lao ethnicity*	74 (59.7)	1.48	0.84–2.60	0.093
	Schooling*	99 (80.5)	1.82	0.945–3.55	0.038
	Student*	8 (6.5)	2.27	0.53–13.6	0.18

*Included in multivariate analysis

**Table 3:** Factors associated with previous wild banana and banana seed consumption and an unawareness of health problems (multivariate analysis) amongst patients (without bowel obstructions) and their relatives at Luang Namtha Provincial Hospital

	Variables	No. (%)	Odds ratio	95% confidence interval	P
Previous wild banana consumption (*n* = 204)	Middle/highland Lao ethnicities	99 (48.8)	9.91	2.23–44.0	0.003
	Male sex	112 (54.9)	4.31	1.50–12.4	0.007
Previous banana seed consumption (*n* = 105)	Middle/highland Lao ethnicities	59 (56.7)	2.33	1.35–4.03	0.002
Unawareness of health problems (*n* = 124)	Male sex	72 (58.1)	1.78	1.05–3.03	0.032

**Table 4:** Traditional methods of treatment of wild banana bowel obstruction as described by patients and relatives visiting the Luang Namtha Provincial Hospital

Treatment	No.	(%)
Poke out stool with stick	31	(30.1
Went to doctor	20	(19.4
Operation	12	(11.7)
Put a (plastic) tube into anus	9	(8.7)
Put a bar of soap into anus	7	(6.8)
Herbal and/or traditional medicine (leaves/animal hairs/ashes/banana leaves/water)	5	(4.9)
Treatment by village traditional healer	3	(2.9)
Put a tube of saline water into stomach	2	(1.9)
Went to doctor who put a tube into anus	1	(1.0)
Vomited and got better	1	(1.0)
Ate sour fruit/green tamarind to force stool out	1	(1.0)
Poke out stool with finger	1	(1.0)
Inject gasoline with a syringe into anus for 1 h, then stool came out	1	(1.0)

Forty-four doctors at 38 hospitals were interviewed; one at each provincial/military hospital (31), one or two from the five referral hospitals (8) and five from the capital's main referral hospital. Twenty-five hospitals (66%) offered general abdominal surgery. Those interviewed were: surgeons (34); generalists (7); and anaesthetist, internist or of unknown specialty (1 each). They had worked at their hospitals for a mean (range) of 14.4 (2–36) years. Thirty-three doctors (75%) were aware of the health problems caused by WB, the majority of whom were from hospitals with a general abdominal surgery department (30/31 versus those without 3/10; OR 80.4, 95% CI 7.8–4470, *P* < 0.001). Thirty (68.2%) doctors had treated such patients. In 2009, 46 of 48 (96%) patients seen had required laparotomy. Doctors described patients mainly as young adults (16/30, 53%; teenagers 6/30, 20%; and children 4/30, 13%), male (24/30, 80%) and middleland Lao (18/30, 60%; highland or lowland each 5/30, 17%). All had eaten banana seeds and the patients had been recorded from all 17 Lao provinces. The estimated minimum incidence of BOWB requiring surgery in 2009 was 0.8/100,000 people and the overall incidence of BOWB was estimated to be 6.5/100,000.

## Discussion

These data suggest that bowel obstruction after WB consumption is a widespread but poorly understood public health problem in Laos. Like other recently described neglected public health problems of the Greater Mekong Valley, such as noma,^17^ epilepsy,^18^ toad poisoning,^19^ paragonimiasis,^20^ and beriberi,^21^ BOWB is probably responsible for substantial morbidity and mortality.

We found a country-wide incidence for operative cases that corresponds to about 3% of all causes for intestinal obstructions in the UK^22^, comparable to volvulus.^22–24^ The literature suggests that phytobezoars are unusual causes of bowel obstruction with a frequency of <1% of admissions due to intestinal obstruction in Canada, Malaysia, USA and England.^7,24–26^ However, in areas with predisposing food habits, such as the consumption of prickly pears or persimmon, higher rates of up to 4% have been recorded.^2,27^ Unlike previous reports, we did not find an association of phytobezoars with previous gastric surgery.^2,27–29^ Phytobezoars are more frequent among males^2,30,31^ and men of middle/highland Lao ethnicity in rural areas were the most affected. Their livelihood derives from farming, fishing and hunting and they have less income, worse health indicators and lower education levels than other Lao groups.^14^ WB consumption was associated with a lack of food when they are working in fields before the rice harvest (October/November) when food insecurity is at its highest.^32^ All four of the Lao patients described by Schoeffl *et al.* were young men and their phytobezoars contained banana seeds.^12^ Our data confirm the importance of seed consumption in WB bezoar formation. Patients’ fasting and the uniformity of the Lao phytobezoars,^12^ i.e. not containing other food types, suggest that an important risk factor is consumption on an empty stomach,^4^ a fact that is little known among the Lao population.

Izumi *et al.* demonstrated that the unripe persimmon's soluble shibuol, a phlobatannin composed of phloroglucin and gallic acid, is coagulated by gastric acids and acts as a ‘cement’ which glues the fruit skin fragments together as a bezoar.^4^ Peeled persimmons without skin fragments do not cause bezoars. Banana fruits also contain tannins, water-soluble phenolics, which are responsible for the sticky, astringent taste when unripe and can interact with pectins and form insoluble complexes.^33^ During ripening, cultivated bananas lose their astringent taste due to increased polymerization and inactivation of tannins.^33,34^ However, unlike cultivated bananas, ripe WB appear to keep their astringency. Our patients reported a sweet but astringent taste indicating the persistence of active tannins.

The existence of traditional treatments suggests that BOWB is not a new disease but one that has been neglected in the scientific literature. Some treatments, such as the poking of stool from the patient’s rectum with sticks, are dramatic and are likely to lead to severe complications such as rectal perforation. Colleagues reported patients with BOWB requiring surgery in northern and southern Vietnam, Cambodia and Myanmar but WB seeds are not eaten by the Karen of eastern Burma because of the risk of constipation (K Mounlaphome, S Phomdouangsi, J Hofmeister, P Mar Soe, H Tran Tinh, Colley Paw Nosten, personal communication).

Our study has the important limitations of retrospective interview surveys. The number of patients is relatively small and the incidence is only estimated from hospital-based data. Those questioned about their knowledge of banana consumption were not chosen at random and usually only one surgeon was interviewed per hospital. We did not investigate BOWB in the large areas of Laos that were without accessible abdominal surgery. Therefore, the incidence was probably considerably underestimated. As relatively few hospitals in Laos offer abdominal surgery, it is likely that many patients in remote Laos with BOWB do not have access to laparotomy.

## Conclusions

BOWB in rural Asia is probably more common than appreciated by those from urban areas and may represent a neglected poverty-related health problem among rural populations in many areas of Asia where WB grow.^10^ Raising the awareness of the importance of not eating WB seeds, especially among the younger male rural population, may help prevent this potentially life-threatening and not uncommon complication. Doctors should consider phytobezoar in the differential diagnosis of bowel obstruction in areas where WB are consumed.
